# Chitosan from Marine Amphipods Inhibits the Wilt Banana Pathogen *Fusarium oxysporum* f. sp. Cubense Tropical Race 4

**DOI:** 10.3390/md21120601

**Published:** 2023-11-22

**Authors:** Marc Roig-Puche, Federico Lopez-Moya, Miguel Valverde-Urrea, Pablo Sanchez-Jerez, Luis Vicente Lopez-Llorca, Victoria Fernandez-Gonzalez

**Affiliations:** 1 Laboratory of Plant Pathology, Department of Marine Sciences and Applied Biology, University of Alicante, 03690 Alicante, Spain; marcpuche9919@gmail.com (M.R.-P.); mrvu1@gcloud.ua.es (M.V.-U.); lv.lopez@ua.es (L.V.L.-L.); 2 Laboratory of Marine Biology, Department of Marine Sciences and Applied Biology, University of Alicante, 03690 Alicante, Spain; psanchez@ua.es (P.S.-J.); victoria.fernandez@ua.es (V.F.-G.)

**Keywords:** amphipods, biofouling, fish farms, chitooligosacharides, Fourier transform infrared spectroscopy (FTIR), Raman, plant pathogenic fungi, banana disease, nematophagous fungi, *Pochonia chlamydosporia*

## Abstract

In this work, we extracted chitosan from marine amphipods associated with aquaculture facilities and tested its use in crop protection. The obtained chitosan was 2.5 ± 0.3% of initial ground amphipod dry weight. The chemical nature of chitosan from amphipod extracts was confirmed via Raman scattering spectroscopy and Fourier transform infrared spectroscopy (FTIR). This chitosan showed an 85.7–84.3% deacetylation degree. Chitosan from biofouling amphipods at 1 mg·mL^−1^ virtually arrested conidia germination (ca. sixfold reduction from controls) of the banana wilt pathogenic fungus *Fusarium oxysporum* f. sp cubense Tropical Race 4 (FocTR4). This concentration reduced (ca. twofold) the conidia germination of the biocontrol fungus *Pochonia chlamydosporia* (Pc123). Chitosan from amphipods at low concentrations (0.01 mg·mL^−1^) still reduced FocTR4 germination but did not affect Pc123. This is the first time that chitosan is obtained from biofouling amphipods. This new chitosan valorizes aquaculture residues and has potential for biomanaging the diseases of food security crops such as bananas.

## 1. Introduction

Since the publication of the Blue Growth strategy in 2012 by the European Commission [1], marine environments have been recognized as potential sources for innovation, economic growth, and job creation. This new sea economy aims to optimize the benefits of sustainably developing marine and maritime sectors [1]. Thus, aquaculture plays a key role in meeting our demand for fish and seafood of high nutritional value [2].

Offshore aquaculture has steadily increased in the marine environment over the last five decades. There is a need to address the challenges posed by this fast growth to provide strategies for the long-term sustainability and conservation of marine ecosystems [3]. The intensity of fish farm exploitation generates byproducts (waste). These wastes may accumulate on marine sediments and cause eutrophication [4]. Integrated multi-trophic aquaculture (IMTA) systems are devised to reduce these environmental impacts. IMTA systems co-culture species from different trophic levels, so by-products from a species become inputs for another [2,5]. Marine species that grow in aquaculture facilities forming biofouling communities can feed on fed waste, contributing to bioremediation [6]. Highly abundant crustacean amphipods in fish farms are suitable for co-culturing in IMTA systems [7].

The circular economy complements Blue Growth, aiming to create a restorative or regenerative industrial system through intention and design, replacing the concept of end-of-life with restoration [8]. It is based on an environmentalist mindset and proposes to change the motto “reduce, reuse and recycle” for a deep and lasting transformation [9]. The new paradigm is to turn waste into raw materials [10]. Thus, marine species that naturally grow in aquaculture facilities could be harvested and used to develop new biological products with applications in several industries, meaning an extra source of income [7].

Marine organisms are a new source of natural products, some with unusual chemical structures [11]. Chitin, one of the most abundant polymers in nature [12], can be found in marine crustaceans, insects, and fungi. Chitosan is a by-product of the partial deacetylation of chitin, primarily consisting of beta-1,4-glucosamine subunit polymers [13]. Chitosan is mostly obtained from by-products of fishing industries; it is a renewable, non-toxic, non-allergenic, antimicrobial, and biodegradable biopolymer [14]. Shellfish waste contains 14–35% chitin associated with proteins (30–40%), lipids, pigments, and calcium deposits (30–50%), and various technological alternatives have been developed for converting shellfish into useful products [15]. As a result, the large biomass of amphipods in aquaculture facilities could be used to produce chitin and chitosan, providing added value to fish farms.

Chitosan has countless applications, including cosmetics, medicine, and agriculture [13,16]. Chitosan from crustaceans has more antimicrobial properties than important worldwide plant pathogens such as *Fusarium oxysporum* or *Magnaporthe oryzae* [17,18,19,20]. Chitosan permeabilizes the plasma membrane of sensitive fungi, with membranes enriched in unsaturated free fatty acids. In contrast, biocontrol fungi (BCA) (mostly nematophagous and entomopathogenic fungi) tolerate chitosan. These BCAs develop infective structures on the exoskeletons and cuticles of their hosts. These structures are enriched in chitin and chitosan [21,22]. BCAs have glycosyl hydrolase families, mainly GH18 (chitinases) and GH 75 (chitosanases) expanded in their genomes [23,24]. These GHs can degrade chitin and chitosan.

In this study, we evaluated marine amphipods associated with aquaculture facilities as a new source of chitosan and its biotechnological use. Our aims are to: (a) extract chitin from amphipods and its conversion to chitosan, (b) characterize amphipod chitosan, and (c) test chitosan from amphipods on the spore germination of agriculturally important fungi: *Pochonia chlamydosporia*, a nematophagous fungus used in biological control; and *Fusarium oxysporum* f. sp. cubense Tropical Race 4 (FocTR4), an emerging and highly virulent fungal pathogen of banana plants.

## 2. Results

### 2.1. Chitosan Extraction

Amphipod exoskeletons contain chitin that can be transformed into chitosan. Table 1 shows chitin/chitosan yields from amphipod samples. Biofouling amphipod’s chitosan yield (2.55 ± 0.34% ground amphipod dry weight) was similar to that from commercial turtle feed amphipods (2.43%). This was despite ca. 50 times more starting materials in the latter. Chitin yields were higher since this biopolymer is the source for chitosan extraction.

### 2.2. Spectroscopical Characterization of Chitin and Chitosan

**Figure 1:**
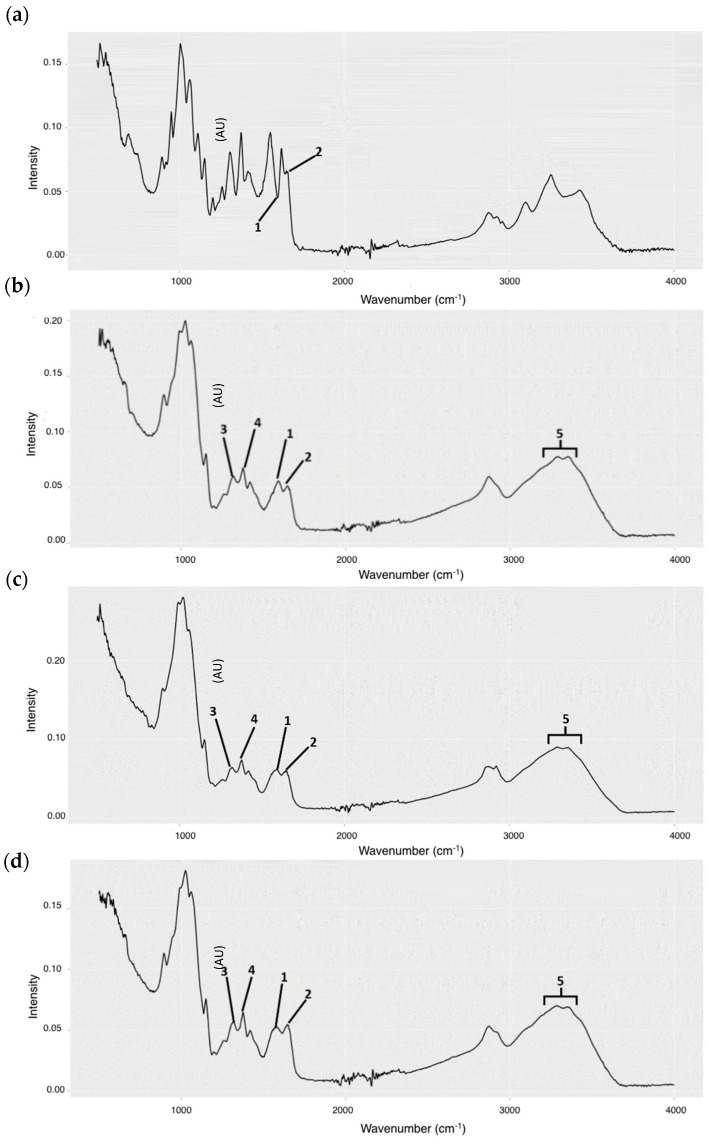
FTIR absorption spectra of chitin and chitosan: (**a**) Commercial chitin; (**b**) Commercial chitosan; (**c**) Biofouling amphipod chitosan (M4); and (**d**) Commercial amphipod (turtle feed) chitosan (M5). Group diagnostic bands: (1) NH_2_ groups (1590 cm^−1^); (2) residual N-acetyl groups (1650 cm^−1^); (3) C–N amide III bond (1320 cm^−1^); (4) CH_3_ groups (1420 cm^−1^); and (5) N–H amide I and O–H pyranose ring bonds (3200–3400 cm^−1^). Intensity is measured in absorbance units (AU).

The chitin–chitosan Raman spectra from the 1500–1800 cm^−1^ region showed a fluorescence background, with both lasers tested (785 and 1064 nm). They were, therefore, discarded. FTIR detected NH_2_ (1) and N-acetyl groups (2), diagnostic for chitin (Figure 1a) and chitosan (Figure 1b–d) in all samples. This confirms that all samples analyzed were either chitin or chitosan. However, in chitin spectra (a), the intensity of band 1 (0.056) was much lower than band 2 (0.096). In contrast, there was little difference between the intensities of both bands for the rest of the samples (chitosan; b1: 0.056, b2: 0.051; c1: 0.062, c2: 0.060; and d1: 0.052, d2: 0.054). The spectra indicate that the b, c, and d samples underwent partial deacetylation, whereas sample a did not. The intensities corresponding to the N–H bonds of amide I and the O–H bond associated with the pyranose ring (5) were only clearly observed in the b, c, and d spectra, indicating that the analyzed samples correspond to chitosan molecules. These bands are the most representative of this biopolymer. For sample a, this region was not observed with the same intensity, suggesting that the spectrum corresponds to chitin.

#### Deacetylation Degree (%DD)

Figure 1 (FTIR spectra) shows the intensity (in AU) of peaks corresponding to the NH_2_ groups and the C–N bond of amide III (peaks 3 and 4, respectively). The %DD of chitosan extracted from amphipods and commercial chitosan (T8) were calculated from them (Table 2). All DDs were above 84%, confirming that these samples are chitosan.

### 2.3. Effect of Chitosan on Spore Germination

#### 2.3.1. *Pochonia chlamydosporia* Isolate 123

**Figure 2:**
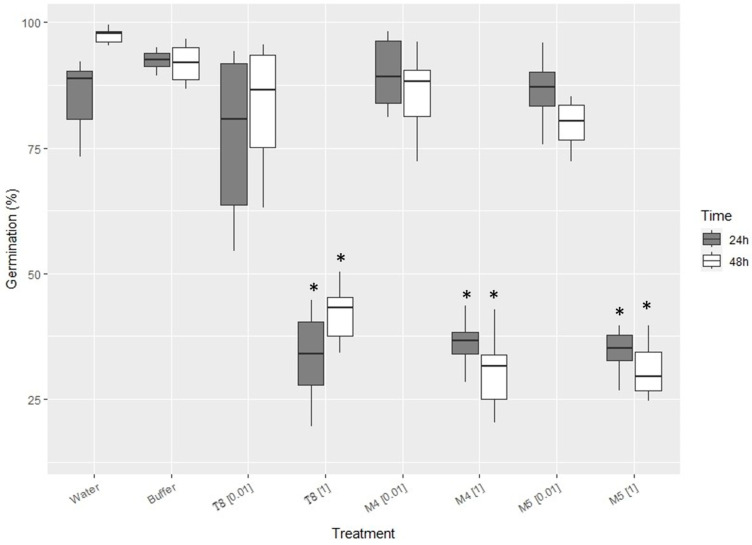
Effect of chitosan from biofouling amphipods and other sources on conidia germination of the nematophagous fungus *P. chlamydosporia*. Treatments: (T8) Commercial chitosan; (M4) Chitosan from biofouling amphipods; (M5) Chitosan from commercial (turtle feed) amphipods. (*) indicates significant differences from controls (*p* < 0.05).

Chitosan from the biofouling amphipods and other sources had no significant (*p*-value < 0.05) effect on the conidia germination of the biocontrol fungus Pc123 at low concentrations (0.01 mg·mL^−1^) (Figure 2). However, all chitosan samples significantly reduced (ca. twofold) Pc123 conidia germination at 1 mg·mL^−1^. No differences (*p*-value *<* 0.05) in *P. chlamydosporia* conidia germination were found for 1 mg·mL^−1^ chitosan from the three sources tested after 24 h and 48 h.

#### 2.3.2. *Fusarium oxysporum* f. sp. cubense Tropical Race 4

**Figure 3:**
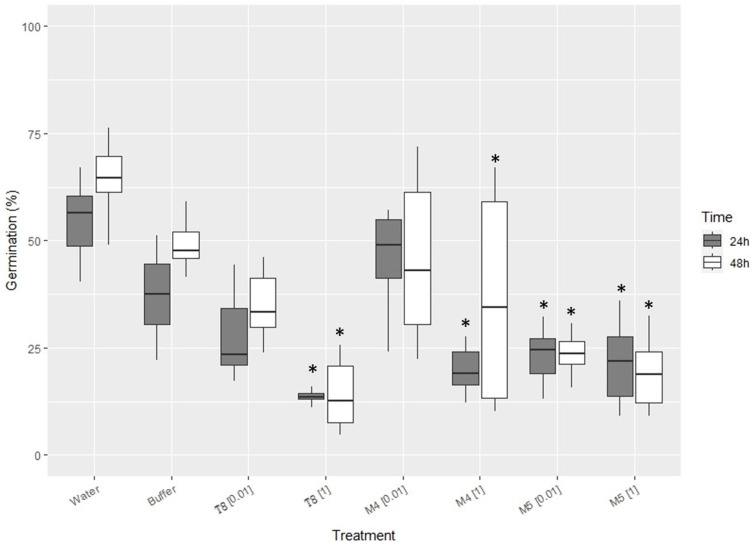
Effect of chitosan from biofouling amphipods and other sources on conidia germination of the phytopathogenic fungus FocTR4. Treatments: (T8) Commercial chitosan; (M4) Chitosan from biofouling amphipods; (M5) Chitosan from commercial (turtle feed) amphipods. (*) indicates significant differences from controls (*p* < 0.05).

Chitosan from biofouling amphipods and other sources at 1 mg·mL^−1^ virtually arrested the conidia germination (ca. sixfold reduction from controls, Figure 3) of the banana wilt pathogenic fungus FocTR4. Most chitosan reduced FocTR4 conidia germination even at a low concentration (0.01 mg·mL^−1^). For *P. chlamydosporia,* no significant (*p*-value < 0.05) differences in FocTR4 spore germination were found for the tested three sources of chitosan.

## 3. Discussion

In our study, we have extracted and characterized chitosan from amphipods associated with biofouling in aquaculture facilities. Chitosan is often obtained from the exoskeletons of marine organisms, mainly crustaceans [25]. This uses waste from the seafood industry via a circular economy approach [15]. However, the current work is, to the best of our knowledge, the first report using amphipods associated with integrated aquaculture as a source for chitosan extraction.

The chitosan extraction yield from amphipods (2.2–2.8%) is significantly lower than that from the exoskeletons of larger crustaceans (e.g., crab, shrimp, or crayfish) [25]. Extraction yields ranging from 9.2 to 22.9% of the initial dry weight are reported, depending on the organism used as raw material.

Raman scattering spectroscopy can provide information on the chemical structures and physical shapes of molecules using characteristic spectral patterns [26]. However, sample fluorescence can be a major issue [27]. In this work, we have experienced that problem. Despite testing several lasers, we were unable to identify this polysaccharide in amphipod extracts using Raman spectroscopy. Fourier transform infrared spectroscopy (FTIR) is the most widely used spectroscopy technique to study vibration in molecules that avoids fluorescence problems. FTIR has allowed us to identify chitosan from amphipod samples. In our analyses, we detected the characteristic wavelengths [28], corroborating the fact that the amphipod extractions contained chitosan or chitin.

The essential parameter for chitosan characterization is the degree of N-deacetylation, determining the physiological and functional characteristics of the biopolymer [29]. Using FTIR data, we have estimated more than 80% of the degree of N-deacetylation of chitosan from biofouling amphipods and other sources, including commercial samples.

In our study, we have found that the conidia germination of the biocontrol fungus Pc123 is unaffected by low concentrations of chitosan from biofouling and commercial-fed amphipods. This agrees with previous studies using chitosan from large crustaceans [30]. However, 1 mg·mL^−1^ causes a reduction from 100% of spore germination to ca. 50% of spore germination, as described using chitosan from crustaceans [30]. *P. chlamydosporia* is a chitosan-resistant fungus. Chitosan increases growth, sporulation, and pathogenicity to plant parasitic nematodes of this fungus [30,31,32,33]. On the other hand, our results indicate that chitosan from biofouling amphipods at 1 mg·mL^−1^ can inhibit the germination of FocTR4, a wilt fungus highly pathogenic on bananas. According to our data, FocTR4 conidia germination seems more resistant than other *Fusarium* wilt pathogens, such as *Fusarium oxysporum* f. sp radicis-lycopersici, a tomato pathogen [33]. Chitosan treatment, applied as a soil drench, showed protection against several wilt diseases, including that caused by different *Fusarium* species on tomatoes [33]. In a recent study, chitosan inhibited *Fusarium spp*. involved in potato wilt [34]. In contrast, the biocontrol fungus Pc123 is tolerant to chitosan concentrations (0.01 mg·mL^−1^), which significantly reduces the germination of Foc TR4. These results suggest that the combination of chitosan and biocontrol agents can help manage disease sustainably in banana and other crops. This has been shown for plant parasitic nematodes [31]. Chitosan has also been described as a plant defense elicitor inducing the expression of salicylic and jasmonic acid pathways in plants [35]. Our results on a new source of chitosan and its application with biocontrol agents and against pathogens and for plant defense induction could open new possibilities for its use in agriculture and other markets.

## 4. Conclusions

In this study, we promote the IMTA system, an environmentally friendly aquaculture procedure. We suggest using amphipods as secondary species to transform them into chitosan, a biopolymer used in numerous fields (e.g., medicine, industry, and agriculture). Chitosan extraction from amphipods is a good approach to valorizing biofouling from fisheries. This chitosan, from a new source, should be tested in future studies for applications that do not require large quantities, such as biomedicine. Methods for chitosan extraction from amphipods should be optimized to maximize yields. However, at present, our results show that chitosan, obtained from amphipods associated with biofouling of aquaculture facilities, can arrest germination—and, therefore, the pathogenicity—of the wilt fungus FocTR4. This virulent new banana pathogen is expanding worldwide, but there are no efficient management methods. Ultimately, amphipod chitosan can help global food security. Our study, thus, contributes to economic sustainability via a new source of income for seafood and aquaculture (fish farms) companies, opening possibilities to generate blue and green circular economy industries.

## 5. Materials and Methods

### 5.1. Sample Collection and Preparation

Samples were collected from a fish farm in the coastal waters of Murcia (Spain, 37°48.93′ N/0°41.73′ W) by scraping fouling organisms from shallow mooring ropes (1–10 m depths). Amphipods were extracted by introducing fouling samples into containers with fresh water for 3 min. Then, they were sieved through a 500 μm mesh and preserved in 70% ethanol. In the laboratory, the subsamples of at least 20% of the amphipod biomass samples were sorted, and amphipods were identified at a species level. The remaining samples were used for chitin/chitosan extraction and characterization. The species composition of amphipod samples is described in Table 3. We have also used amphipods from turtle foods (*Gammarus* spp. for turtle feed, Terra Viva) for comparison purposes.

### 5.2. Chitosan Extraction

Amphipod samples were dried at room temperature for 4 h and crushed in a mortar with liquid nitrogen until a homogeneous powder was obtained. Then, the sample was demineralized using a 0.5 M hydrochloric acid at a 1:30 *w*/*v* ratio for 2 h at room temperature under constant stirring [36]. The sample was washed with deionized H_2_O to pH 7 and left to dry at 60 °C for 6 h. Protein removal was performed in a 0.5 M sodium hydroxide with a *w*/*v* 1:40 for 19 h at room temperature under constant stirring. The chitin obtained was washed with deionized H_2_O to pH 7 and left to dry in an oven at 60 °C for 6 h. Chitin was bleached by shaking it in 2% sodium hypochlorite [25] at a 1:100 *w*/*v* ratio for 10 min. The sample was washed with deionized H_2_O to neutral pH and left to dry in an oven at 60 °C for 6 h. Chitin was deacetylated with 50% sodium hydroxide in a 1:100 *w*/*v* ratio at 100 °C for 2 h under constant stirring [37]. Finally, the obtained chitosan was washed with deionized H_2_O to a pH of 7 and dried at 60 °C for 6 h.

### 5.3. Chitosan Characterization

Commercial chitosan (Marine BioProducts GmbH, Rosenheim, Germany), chitin (Sigma-Aldrich, St. Louis, MO, USA), and chitosan obtained from amphipods were subjected to Raman scattering spectroscopy, Fourier transform infrared spectroscopy (FTIR), and degree of deacetylation (%DD) calculation.

Raman scattering spectroscopy was used to detect the bond vibrations of the functional groups that make up chitosan and chitin samples to provide information on their chemical structures and physical forms [26]. NRS5100 Raman spectrometer was used. Both 785 nm and 1064 nm lasers were used. The latter usually generated fewer fluorescence emission artifacts. A study of the whole spectrum and another at the 1500–1800 cm^−1^ region, characteristic for differentiating chitin and chitosan, were carried out (Prof. A. C. Prieto, University of Valladolid, Spain. Personal communication).

FTIR analysis was carried out to characterize the obtained chitosan and compare them with chitin and commercial chitosan samples. This technique is based on the excitation of the molecules to be studied to observe the infrared absorption of the bonds they present, assigning the peaks corresponding to the bending and/or stretching of the bonds at wavelengths (in cm^−1^) characteristic of the molecules under study. The characteristic bands used for chitosan are in the 3200–3400 cm^−1^ region, corresponding to the N–H bond of amide I and the O–H bond associated with the pyranose ring, respectively [38]. The 1650 cm^−1^ bands corresponding to the residual N-acetyl groups and the 1590 cm^−1^ corresponding to the NH_2_ groups were analyzed to differentiate the chitin and chitosan spectra [39]. When chitin is deacetylated, the intensity of the 1650 cm^−1^ bands decays, while the 1590 cm^−1^ band increases, indicating that the acetyl groups have been hydrolyzed and substituted by NH_2_ groups [40]. Infrared spectra studies were carried out using the FT/IR-4700Type spectrophotometer (BRUKER, Billerica, MA, USA). The measurements were performed using the ATR PRO ONE mode of operation at an incidence angle of 45º, in the range of 500–4000 cm^−1^. The number of scans was 3632, accumulating 16 scans per scan point, with a resolution of 4 cm^−1^.

Chemically, chitin and chitosan are polyglucosamines distinguished only by the degree of acetylation, or the degree of deacetylation (one inverse of the other), of the amino groups. Chitin usually has a degree of deacetylation between 5 and 30% [28], whereas the %DD of chitosan must be greater than 50% to be considered such a molecule [41]. This parameter is one of the most important to determine the functional and physiological characteristics of the polymer. The spectra obtained in the FTIR study were used to calculate N-acetylation. The values obtained in the characteristic bands of amide III (1320 cm^−1^) and, as a reference, the methyl groups (1420 cm^−1^) were taken. Calculations of the degree of N-acetylation were obtained from the following equation [28]:

$$\%\;Degree\;of\;N-acetylation=31.92\times \frac{A_{1320}}{A_{1420}}-12.2$$

The degree of N-deacetylation (DD) of chitin or chitosan is the complementary value demonstrated as follows [42]:

% DD=100−Degree of N−acetylation

### 5.4. Preparation of Chitosan Solutions

Chitosan from a given source was dissolved in 0.25 M HCl under continuous stirring to obtain an initial concentration of 10 mg·mL^−1^, and pH was adjusted to 5.6 with 1 M and 0.1 M NaOH [17]. The obtained solution was dialyzed for salt removal for 48 h. The dialyzed chitosan was autoclaved (120 °C, 20 min) before use. We also prepared a 0.25 M HCl solution and adjusted it to pH 5.6 using 1 M and 0.1 M NaOH. This solution was dialyzed and autoclaved for chitosan [17].

### 5.5. Effect of Chitosan on Spore Germination

The fungal material used was strain Pc 123 of the nematophagous fungus *Pochonia chlamydosporia* 123 (Pc123, ATCC No. MYA-4875; CECT No.20929) isolated from eggs of *Heterodera avenae* in Seville. A further fungus used was a strain of *Fusarium oxysporum* f. sp. cubense Tropical Race 4 (FocTR4) (E.F. Smith) Snyder and Hansen, NRRL36114 (CBS 102025), isolated from the hybrid (*Musa acuminata* (AA) × *M. balbisiana* (BB)) *Pisang Manurung* (AAB) in Indonesia. The strain was acquired from the Institute of Fungal Biodiversity Westerdijk NL (authorization MiPAAF 31519, dated 6 December 2017), a former CBS collection of the Netherlands. Fungi were routinely grown on potato dextrose agar (PDA, Oxoid, Basingstoke, Hampshire, UK).

Conidia used for these experiments were collected from 2-week-old cultures of fungi growing in the media described above. The conidia were collected from the plates with 1 mL sterile distilled water, passed through a glass wool filter to remove hyphae, counted, and diluted to 1 × 10^6^ conidia·mL^−1^.

Three chitosan sources were used for experiments: commercial chitosan (T8, Biolog Heppe, GmbH Landsberg, Bremen, Germany), chitosan from aquaculture amphipods extracted as before (M4), and chitosan extracted for commercial amphipods (freeze-died natural *Gammarus* spp. for turtle feed, Terra Viva) (M5).

We designed moist chamber experiments in 9 cm diameter Petri dishes with autoclaved filter paper moistened with 1 mL of autoclaved distilled water to observe the effect of chitosan on Pc123 and FocTR4 conidia. We used 10 µ/L droplets with a concentration of 1 × 10^6^ conidia·mL^−1^ with chitosan at either 0.01 or 1 mg·mL^−1^ on autoclaved microscopic glass slides. Controls consisting of autoclaved distilled water or HCl–NaOH buffer pH = 5.6 were also prepared.

For each treatment, 6 plates were prepared with 3 drops each. Three of the plates were incubated at room temperature and under 16 h light/8 h dark for 24 h and the other three for 48 h. After incubation, drops on slides were observed under an Olympus BH2 microscope (Olympus, Tokyo, Japan) with a Leica DFC 480 camera (Leica, Wetzlar, Germany). Observations were made at 20× magnification, observing a random field (with a total number of conidia of 200–800, approx.) and counting all germinated and non-germinated conidia, thus obtaining a germination percentage for each treatment and time.

### 5.6. Statistical Analysis

To analyze the effect of chitosan on the germination of Pc123 and FocTR4, a two-factor ANOVA was performed by considering ‘Time’ (fixed and orthogonal) with two levels (24 h and 48 h) and ‘Treatment’ (fixed and orthogonal) with four levels (H_2_O, Buffer, chitosan concentration (0.01) and (1) mg·mL^−1^) on 3 samples to every level for each of the three-chitosan studied. The dependent variable was the germination percentage. Normality was tested with the Kolmogorov–Smirnov test. If normality was not met, as this study was balanced and *n* > 30, ANOVA was considered sufficiently robust. The homogeneity of variances was tested with Barlett’s test. Data were transformed if necessary. However, if the homogeneity of variances was not met, a *p*-value of α < 0.01 was considered significant. If significant differences were found in the ANOVA, an a posteriori test (Tukey HSD) was performed. All statistical analyses were carried out with the free software R v 4.1.2 [43].

## Figures and Tables

**Table 1:** Chitin and chitosan purification from amphipods in this work.

Step	Sample Weight (g)				
	M1	M2	M3	M4	M5
Ground-dried amphipods	1.205	1.302	1.188	14.1	49.31
Demineralization	0.416	0.56	0.469	5.45	20.52
Deproteinization	0.156	0.151	0.121	2.955	2.85
Chitin	0.093	0.083	0.064	0.983	2.024
Chitosan	0.034	0.037	0.027	0.312	1.198
Chitin Yield (%)	7.72	6.37	5.39	6.97	4.1
Chitosan Yield (%)	2.82	2.84	2.27	2.21	2.43
Biofouling Amphipods (M1–M4) Chitin Yield (% ± SD)			6.61± 0.98		
Biofouling Amphipods (M1–M4) Chitosan Yield (% ± SD)			2.55 ± 0.34		

M1–4 chitosan from biofouling aquaculture amphipods. M5 chitosan from commercial amphipods (turtle food). SD indicates the standard deviation (in %) from the mean yield of chitin and chitosan in the biofouling aquaculture amphipod samples (M1–M4).

**Table 2:** Chitosan N-deacetylation degree (DD).

Chitosan Source	%DD
T8	85.2–(90.1 *)
M1	85.7
M2	85.3
M3	85.3
M4	84.3
M5	83.3

* %DD from (DD) data sheet provided by the manufacturer.

**Table 3:** Taxonomic composition of amphipods causing biofouling.

Genus/Species	Percentage (%)
*Jassa* spp.	59.7
*Ericthonius punctatus*	27.7
*Elasmopus rapax*	5.5
*Stenothoe* spp.	5
*Caprella equilibra*	2.3

## Data Availability

The data that support the findings of this study are available from the corresponding author upon reasonable request.

## References

[1] Lillebø A.I., Pita C., Rodrigues J.G., Ramos S., Villasante S. (2017). How can marine ecosystem services support the Blue Growth agenda?. Mar. Policy.

[2] FAO Agriculture Organization of the United Nations Fisheries Department. The State of World Fisheries and Aquaculture, 2014 (Volume 3). Food and Agriculture Org. https://www.fao.org/3/i3720e/i3720e.pdf.

[3] COM/2021/236 Final (2021). Strategic Guidelines for a More Sustainable and Competitive EU Aquaculture for the Period 2021 to 2030. Communication from the Commission to the European Parliament, the Council, the European Economic and Social Committee and the Committee of the Regions. https://eur-lex.europa.eu/legal-content/ES/TXT/HTML/?uri=CELEX:52021DC0236&from=EN.

[4] Kalantzi I., Karakassis I. (2006). Benthic impacts of fish farming: Meta-analysis of community and geochemical data. Mar. Pollut. Bull..

[5] Rosa J., Lemos M.F., Crespo D., Nunes M., Freitas A., Ramos F., Leston S. (2020). Integrated multitrophic aquaculture systems–Potential risks for food safety. Trends Food Sci. Technol..

[6] Gonzalez-Silvera D., Izquierdo-Gomez D., Fernandez-Gonzalez V., Martínez-López F.J., López-Jiménez J.A., Sanchez-Jerez P. (2015). Mediterranean fouling communities assimilate the organic matter derived from coastal fish farms as a new trophic resource. Mar. Pollut. Bul..

[7] Fernandez-Gonzalez V., Toledo-Guedes K., Valero-Rodriguez J.M., Agraso M.D.M., Sanchez-Jerez P. (2018). Harvesting amphipods applying the integrated multitrophic aquaculture (IMTA) concept in off-shore areas. Aquaculture.

[8] MacArthur E. (2014). Towards the Circular Economy: Accelerating the Scale-Up across Global Supply Chains.

[9] Lett L.A. (2014). Las amenazas globales, el reciclaje de residuos y el concepto de economía circular. Rev. Argent. Microbiol..

[10] Martínez-Vázquez R.M., Milán-García J., de Pablo Valenciano J. (2021). Challenges of the Blue Economy: Evidence and research trends. Environ. Sci. Eur..

[11] König G.M., Kehraus S., Seibert S.F., Abdel-Lateff A., Müller D. (2006). Natural products from marine organisms and their associated microbes. ChemBioChem.

[12] Cohen-Kupiec R., Chet I. (1998). The molecular biology of chitin digestion. Curr. Opin. Biotechnol..

[13] Kumar M.N.V.R. (2000). A review of chitin and chitosan applications. React. Funct. Polym..

[14] Pillai C.K.S., Paul W., Sharma C.P. (2009). Chitin and chitosan polymers: Chemistry, solubility and fiber formation. Prog. Polym. Sci..

[15] Mármol Z., Páez G., Rincón M., Araujo K., Aiello C., Chandler C., y Gutiérrez E. (2013). Quitina y Quitosano polímeros amigables. Una revisión de sus aplicaciones/Chitin and Chitosan friendly polymer. A review of their applications. Rev. Tecnocientífica URU.

[16] Zhao D., Yu S., Sun B., Gao S., Guo S., Zhao K. (2018). Biomedical applications of chitosan and its derivative nanoparticles. Polymers.

[17] Palma-Guerrero J., Jansson H.B., Salinas J., López-Llorca L.V. (2008). Effect of chitosan on hyphal growth and spore germination of plant pathogenic and biocontrol fungi. J. Appl. Microbiol..

[18] Al-Hetar M.Y., Zainal Abidin M.A., Sariah M., Wong M.Y. (2011). Antifungal activity of chitosan against *Fusarium oxysporum* f. sp. cubense. J. Appl. Polym. Sci..

[19] Lopez-Moya F., Martin-Urdiroz M., Oses-Ruiz M., Were V.M., Fricker M.D., Littlejohn G., Lopez-Llorca L.V., Talbot N.J. (2021). Chitosan inhibits septin-mediated plant infection by the rice blast fungus *Magnaporthe oryzae* in a protein kinase C and Nox1 NADPH oxidase-dependent manner. New Phytol..

[20] Ren J., Tong J., Li P., Huang X., Dong P., Ren M. (2021). Chitosan is an effective inhibitor against potato dry rot caused by *Fusarium oxysporum*. Physiol. Mol. Plant. Pathol..

[21] Palma-Guerrero J., Huang I.C., Jansson H.B., Salinas J., Lopez-Llorca L.V., Read N.D. (2009). Chitosan permeabilizes the plasma membrane and kills cells of *Neurospora crassa* in an energy dependent manner. Fungal Genet. Biol. Fungal Genet. Biol..

[22] Palma-Guerrero J., Lopez-Jimenez J.A., Pérez-Berná A.J., Huang I.C., Jansson H.B., Salinas J., Villalaín J., Read N.D., Lopez-Llorca L.V. (2010). Membrane fluidity determines sensitivity of filamentous fungi to chitosan. Mol. Microbiol..

[23] Larriba E., Jaime M.D., Carbonell-Caballero J., Conesa A., Dopazo J., Nislow C., Martín-Nieto J., Lopez-Llorca L.V. (2014). Sequencing and functional analysis of the genome of a nematode egg-parasitic fungus, *Pochonia chlamydosporia*. Fungal Genet. Biol..

[24] Aranda-Martinez A., Lenfant N., Escudero N., Zavala-Gonzalez E.A., Henrissat B., Lopez-Llorca L.V. (2016). CAZyme content of *Pochonia chlamydosporia* reflects that chitin and chitosan modification are involved in nematode parasitism. Env. Microbiol..

[25] Kaya M., Dudakli F., Asan-Ozusaglam M., Cakmak Y.S., Baran T., Mentes A., Erdogan S. (2016). Porous and nanofiber α-chitosan obtained from blue crab (*Callinectes sapidus*) tested for antimicrobial and antioxidant activities. LWT-Food Sci. Technol..

[26] Smith E., Dent G. (2019). Modern Raman Spectroscopy: A Practical Approach.

[27] Agarwal U.P. (2019). Analysis of cellulose and lignocellulose materials by Raman spectroscopy: A review of the current status. Molecules.

[28] Escobar Sierra D.M., Castro Ramírez A.M., y Vergara Castrillón N.A. (2014). Determining the Relation between the Proportion of the Amino Group and the Degree of Deacetylation of Chitosan. Rev. Cienc..

[29] Grifoll-Romero L., Pascual S., Aragunde H., Biarnés X., Planas A. (2018). Chitin deacetylases: Structures, specificities, and biotech applications. Polymers.

[30] Escudero N., Sebastiao R.F., Lopez-Moya F., Naranjo-Ortiz M.A., Marin-Ortiz A.I., Thornton C.R., Lopez-Llorca L.V. (2016). Chitosan enhances parasitism of *Meloidogyne javanica* eggs by the nematophagous fungus *Pochonia chlamydosporia*. Fungal Biol..

[31] Escudero N., Lopez-Moya F., Ghahremani Z., Zavala-Gonzalez E.A., Alaguero-Cordovilla A., Ros-Ibañez C., Lopez-Llorca L.V. (2017). Chitosan increases tomato root colonization by *Pochonia chlamydosporia* and their combination reduces root-knot nematode damage. Front. Plant Sci..

[32] Lopez-Moya F., Suarez-Fernandez M., Lopez-Llorca L.V. (2019). Molecular Mechanisms of Chitosan Interactions with Fungi and Plants. Int. J. Mol. Sci..

[33] Jabnoun-Khiareddine H., El-Mohamed R.S.R., Abdel-Kareem F., Aydi Ben Abdallah R., Gueddes-Chahed M., Daami-Remadi M. (2015). Variation in chitosan and salicylic acid efficacy towards soil-borne and air-borne fungi and their suppressive effect of tomato wilt severity. J. Plant Pathol. Microbiol..

[34] Mejdoub-Trabelsi B., Touihri S., Ammar N., Riahi A., Daami-Remadi M. (2020). Effect of chitosan for the control of potato diseases caused by *Fusarium* species. J. Phytopathol..

[35] Lopez-Moya F., Escudero N., Zavala-Gonzalez E.A., Esteve-Bruna D., Blázquez M.A., Alabadí D., Lopez-Llorca L.V. (2017). Induction of auxin biosynthesis and WOX5 repression mediate changes in root development in *Arabidopsis* exposed to chitosan. Sci. Rep..

[36] Cauchie H.M. (2002). Chitin production by arthropods in the hydrosphere. Hydrobiologia.

[37] Escobar Sierra D.M., Urrea Llano C.A., Gutiérrez Guerra M., y Zapata Ocampo P.A. (2011). Producción de matrices de quitosano extraído de crustáceos. Rev. Ing. Biomédica.

[38] Queiroz M.F., Teodosio Melo K.R., Sabry D.A., Sassaki G.L., Rocha H.A.O. (2014). Does the use of chitosan contribute to oxalate kidney stone formation?. Mar. Drugs.

[39] Kumari S., Rath P., Kumar A.S.H., y Tiwari T.N. (2015). Extraction and characterization of chitin and chitosan from fishery waste by chemical method. Environ. Technol. Innov..

[40] Paulino A.T., Simionato J.I., Garcia J.C., Nozaki J. (2006). Characterization of chitosan and chitin produced from silkworm crysalides. Carbohydr. Polym..

[41] Rinaudo M. (2006). Chitin and chitosan: Properties and applications. Prog. Polym. Sci..

[42] Yen M.T., Yang J.H., Mau J.L. (2009). Physicochemical characterization of chitin and chitosan from crab shells. Carbohydr. Polym..

[43] R Core Team (2020). R: A Language and Environment for Statistical Computing.

